# Evaluation of Approach to a Conspecific and Blood Biochemical Parameters in TAAR1 Knockout Mice

**DOI:** 10.3390/brainsci12050614

**Published:** 2022-05-08

**Authors:** Ilya S. Zhukov, Maria A. Ptukha, Ekaterina A. Zolotoverkhaja, Ekaterina L. Sinitca, Ilya Y. Tissen, Inessa V. Karpova, Anna B. Volnova, Raul R. Gainetdinov

**Affiliations:** 1 Institute of Translational Biomedicine, Saint Petersburg State University, Universitetskaya Nab., 7–9, 199034 Saint Petersburg, Russia; ptukhamaria@yandex.ru (M.A.P.); a.volnova@spbu.ru (A.B.V.); 2 S. Anichkov Department of Neuropharmacology, Institute of Experimental Medicine, Federal State Budgetary Scientific Institution, Academica Pavlova Str. 12, 197376 Saint Petersburg, Russia; sin.e@list.ru (E.L.S.); iljatis@mail.ru (I.Y.T.); inessa.karpova@gmail.com (I.V.K.); 3 Laboratory of Biochemical Toxicology and Pharmacology, Golikov Research Center of Toxicology, Federal Medical-Biological Agency, Bekhtereva Str. 1, 192019 Saint Petersburg, Russia; e.zolotoverkhaja@yandex.ru; 4 Saint Petersburg State University Hospital, Saint Petersburg State University, Universitetskaya Nab., 7–9, 199034 Saint Petersburg, Russia

**Keywords:** trace amines, TAAR, mice sexual motivation, biochemistry, testosterone, TAAR1, safety profile

## Abstract

It is known that the trace amine-associated receptor 1 (TAAR1) receptor is involved in limbic brain functions by regulating dopamine transmission and putative reward circuitry. Moreover, other TAARs are expressed in the olfactory system of all studied vertebrate species, sensing innate socially-relevant odors, including pheromones. Therefore, one can assume that TAARs may play a role in rodent social and sexual behavior. A comparative behavioral and biochemical analysis of TAAR1 knockout (TAAR1-KO) and wild-type mice is also important for the preliminary evaluation of the potential side effects of future TAAR1-based therapies. In our studies, we adapted a sexual incentive motivation test for mice to evaluate the sexual behavior of TAAR1-KO and wild-type mice. Previously, similar methods were primarily applied to rats. Furthermore, we measured testosterone and other biochemical parameters in the blood. As a result, we found only minimal alterations in all of the studied parameters. Thus, the lack of TAAR1 does not significantly affect sexual motivation and routine lipid and metabolic blood biochemical parameters, suggesting that future TAAR1-based therapies should have a favorable safety profile.

## 1. Introduction

In 2001, two independent groups discovered a family of monoamine-related G protein-coupled receptors (GPCRs) named Trace Amine-Associated Receptors (TAARs) [1,2,3]. In mammals, nine subfamilies of trace amine-associated receptors (TAAR1-9) genes are known. Three of them are pseudogenes in humans [4,5]. The classic examples of trace amines are β-phenylethylamine (PEA), p-tyramine (TYR), tryptamine (TRP), and p-octopamine (OCT). Trace amines are structurally and functionally close to classical monoamine neurotransmitters such as dopamine, serotonin, and norepinephrine, but their tissue concentrations are more than 100 times lower [6]. Nowadays, TAAR1 is one of the most investigated trace amine-associated receptors. Its expression was found in the limbic brain areas and certain peripheral tissues. Significant alterations in the brain dopamine, serotonin and glutamate function were found in TAAR1 knockout (TAAR1-KO) mice [4,5]. Based on elevated dopamine transmission, supersensitivity of D2 dopamine receptors and enhanced responsiveness to amphetamine, TAAR1-KO mice were proposed as a model of schizophrenia [4,5]. In fact, the first drug based on TAAR1 agonism has successfully passed Phase II of clinical trials for the treatment of schizophrenia [7].

Previous studies demonstrated that alterations in mesolimbic and mesocortical dopaminergic neurons could affect several aspects of rodent sexual behavior [8,9,10,11,12,13,14,15,16]. Recent studies in dopamine transporter (DAT) knockout rats confirmed a key role of dopamine in sexual behavior. They provided evidence that the permanently elevated dopamine levels triggered by DAT gene silencing can significantly affect male sexual motivation [17]. TAAR1 can modulate the dopamine system via the formation of the TAAR1/D2R heteromer complex [5]. Several lines of evidence indicate that the TAAR1 is involved in limbic networks and can be involved in putative reward functions [4,5]. At the same time, TAAR2-TAAR9 are expressed in the olfactory epithelium of all studied vertebrate species, functioning as sensors of socially-relevant innate odors, including pheromones [18,19,20]. Moreover, recent studies indicate that TAAR5 and likely other “olfactory” TAARs are also expressed in the limbic brain areas and modulate adult neurogenesis [21,22,23]. These observations suggest that such an impact of TAARs on the central nervous system (CNS) functions can potentially affect social and sexual functions. In the present study, we used a non-contact sexual incentive motivation test (SIMT) to evaluate sexual motivation. This male sexual behavior assessment method was used mostly in rats, but we adapted this method for mice. Testosterone (TSTO) is a pivotal hormone involved in regulating male sexual function, acting both at the central and peripheral levels [24]. The analysis of sexual behaviors and TSTO blood levels in mice lacking TAAR1 allows for the evaluation of potential risks related to future TAAR1-based therapies.

Most investigations in the TAAR1 field were focused on brain neurotransmission functions [25,26]. However, TAAR1 is known to also be widely expressed outside of the nervous system. Activated platelets can release PEA and TYR [27]. These compounds chemoattract neutrophils via the TAAR1 and TAAR2 heterodimer complex [28]. TAARs may be generally involved in the process of leukocyte recruitment to the injury sites [5]. At the same time, the lack of TAAR1 and TAAR5 does not lead to significant changes in platelets and other hematological parameters, even in older mice [29,30]. On the other hand, a recent study demonstrated that increased TAAR1 expression in monocytes mediated anti-inflammatory effects in multiple sclerosis [31]. As a result, one can expect a significant role of TAAR1 in immune regulation.

TAAR1 can also be involved in thyroid regulation. Primary cilia (PC) are microtubule-based sensory organelles with various receptors and channels involved in thyroid regulation [32]. The expression of TAAR1 was localized at the PC of thyroid epithelial cells in in vitro and in situ experiments [33]. Further studies demonstrated that the deletion of the TAAR1 gene led to phenotypic changes in thyroid morphology and its functional activity [34]. Furthermore, the visualization of the trafficking of mouse TAAR1 to the cilia of thyroid epithelial cells was performed with a green fluorescent protein [35]. In addition, it was proposed that high TAAR1 expression can be a positive prognosticator for overall survival in ovarian cancer patients [36]. Interestingly, ovarian cancer is regulated by thyroid hormones and their derivatives [37,38]. However, the role of TAARs in the non-canonical regulation of the thyroid system is still unclear, and further studies are needed.

TAAR1 receptor expression was also found in pancreatic β-cells, the stomach, and the intestines [39]. A significant role of TAAR1 and probably other TAARs in type 2 diabetes and obesity was indicated [40]. Thus, such an impact of TAAR1 on the biological system may lead to metabolic and lipid exchange imbalances. Recently, we found significantly decreased low-density lipoprotein cholesterol (LDL-cholesterol) changes in cholesterol levels in TAAR9 knockout rats [41]. Thus, the analysis of routine biochemical parameters in the blood of TAAR1 knockout (TAAR1-KO) mice is of interest. In a previous study, we investigated TAAR1-KO mice to evaluate the safety profile for TAAR1 potential treatments from the perspective of clinical hematology, basic behavioral tests, and thyroid regulation [29]. The current study focused on the sexual motivation behavior of TAAR1-KO mice, fulfilled with additional hormone parameters and biochemical screening.

## 2. Materials and Methods

### 2.1. Animals

All animal studies were carried out according to the Ministry of Health of Russian Federation guidelines and the principles adopted by the FELASA and RusLASA organizations’ welfare of laboratory animal use. All experiments were approved by the Saint Petersburg State University Ethical Committee for Animal Research (No. 131-03-1 of 16 July 2020). Wild-type (WT) and TAAR1-KO mice were derived by crossing (over 20 generations) heterozygous TAAR1 C57BL6/129SvJ animals. Experimental male mice (30 weeks old) and WT female mice (14 weeks old) were housed 3–5/cage, maintained under standard lab conditions (room temperature and humidity were 21 ± 5 °C and 40–70%, respectively), and provided with food and water ad libitum. All experiments were conducted during the light phase. The mice were habituated to the experimental room for at least 1 h before the behavioral experiments.

### 2.2. Sample Collection and Storage

To prepare serum for biochemical screening and automated ELISA, mice were decapitated, and blood was collected into VACUETTE blood collection tubes for serum (Greiner Bio-One, Austria, Kremsmünster), incubated in a vertical position for 15 min, and then kept at +4 °C until centrifugation. Samples with coagulated blood were centrifuged at 1500 rpm for 15 min at +4 °C. Serum was transferred into dry clean tubes and stored until analysis at −20 °C for no more than 3 days.

### 2.3. Measurement of Biochemical Parameters

TAAR1 biochemical screening was performed on automatic analyzer Random Access A-25 (Biosystems S.A., Spain, Barcelona), which was used utilizing the spectrophotometer principle. Serum samples were stored at −20 °C before analysis. The following biochemical parameters were analyzed: alanine aminotransferase (ALT), aspartate aminotransferase (AST), total protein, urea, triglycerides (TG), lactate dehydrogenase (LDH), creatine kinase, alkaline phosphatase (ALP), total cholesterol (TC), low-density lipoprotein cholesterol (LDLC), high-density lipoprotein cholesterol (HDLC), albumin, total bilirubin (TB), creatinine. The full data, number of samples and dilution factors are presented in the Supplementary Materials (Table S1).

### 2.4. Measurement of Testosterone

We used an automatic analyzer based on the ELISA principle, Advia Centaur XP (Siemens Healthineers, Germany, Erlangen), to measure testosterone. Serum samples were diluted with sterile pyrogen-free 0.9% sodium chloride solution in a ratio of 1:3.

### 2.5. Interassay Repeatability

Before analyzing the serum and blood samples, the equipment was decontaminated, calibrated, and checked by a laboratory quality control. Interassay repeatability was evaluated by calculating the coefficient of variation (CV) of 10 consecutive internal quality control material measurements in three different controls: high, normal, and low. CVs were calculated as standard deviation (SD)/mean × 100.

### 2.6. Sexual Incentive Motivation Test Protocol

**Figure 1:**
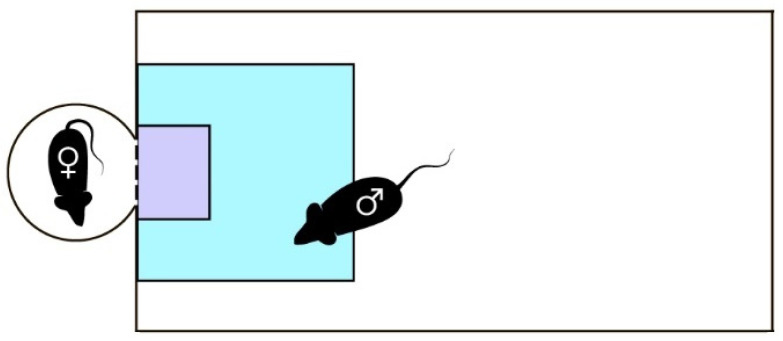
Modified sexual incentive motivation test setup. Setup includes experimental chamber (15 × 30 × 50 cm^3^) and incentive cage separated by a wire-screen (dashed line) from each chamber. White circle with female mouse—incentive cage; blue square—“female” zone, 10 × 10 cm^2^; purple rectangle—“nose” zone, 4 × 3 cm^2^.

1. Hormone-induced estrous. WT female mice in estrous were used as a sexual incentive. To induce estrous, adult female mice were given 10 μg estrogen benzoate and 500 μg progesterone intraperitoneally 48 and 2 h before the experiment, respectively [42]. The stage of the cycle was checked 1 h before the experiment via an assessment of vaginal smears [43].
2. Experimental setup and analysis. A modified sexual incentive motivation test (SIMT) was used to evaluate the sexual behavior of TAAR1-KO and WT male mice [44]. The setup consisted of 4 experimental chambers (15 × 30 cm^2^), each with an adjacent incentive cage separated by a permeable wall (Figure 1). A female mouse in estrous was placed in the incentive cage 20 min before the experiment. A male mouse was then placed into the experimental chamber for 20 min, while the behavior was recorded and then processed using Noldus EthoVision XT (Version 11.5; Noldus Information Technology, Wageningen, The Netherlands). To speed up the data collection and analysis, a set-up of 4 such independent cages was used simultaneously.
3. To assess the recognition of sexually relevant stimuli, two zones were differentiated for the analysis: a 10 × 10 cm^2^ zone adjacent to the incentive cage called “female”, and a 4 × 3 cm^2^ zone closest to the cage called “nose”. The following parameters potentially descriptive of sexual behavior were analyzed: percentage of time spent in the “female” zone by the center-point of the animal’s body, number of visits to the “female” zone by the center-point, percentage of time spent in the “nose” zone by the nose-point, number of visits to the “nose” zone by the nose-point.
4. Experimental design. Firstly, to assess the validity of the suggested method, 15 WT male mice were tested in two conditions: in the presence of a sexual incentive and with empty incentive cages. The parameters described in the previous paragraph were compared to assess the validity of individual parameters and the method itself for the evaluation of sexual behavior. 16 TAAR1-KO and 15 WT male mice were tested in SIMT.

### 2.7. Statistical Analysis

In the SIMT, a two-way analysis of variance (ANOVA) with repeated measures was used to compare all data, which was preliminarily tested for Gaussian distribution with the D’Agostino-Pearson normality test. Hormonal and biochemical parameters data between two groups were analyzed using a non-parametric Mann–Whitney test. Analyses were performed using GraphPad Prizm 8 (GraphPad Software, San Diego, CA, USA).

## 3. Results

### 3.1. Lack of TAAR1 Does Not Affect Sexual Motivation

**Figure 2:**
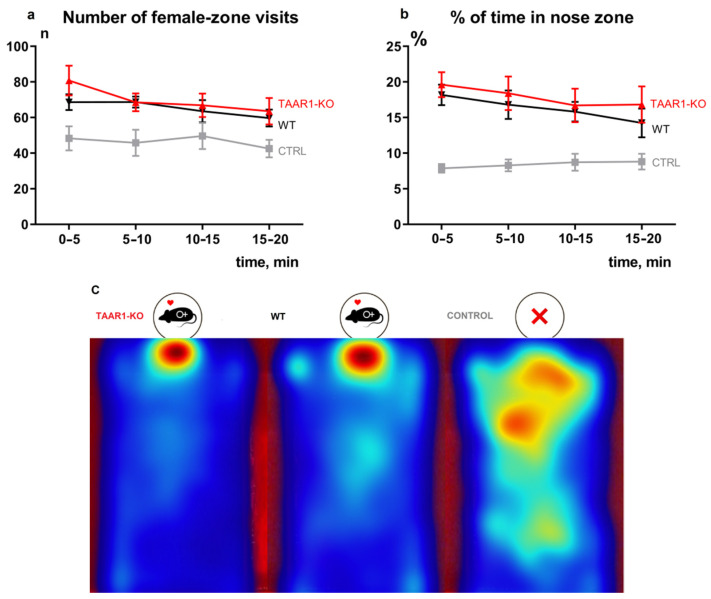
SIMT behavior parameters panel for TAAR1-KO and WT mice. No alterations in sexual incentive behavior in TAAR1-KO male mice in the SIMT test. (**a**) There are no significant differences in the number of visits to the female zone (**b**) and the percent of the time in the nose zone between WT and TAAR1-KO mice. All other behavioral parameters also revealed minimal alterations. Additionally, the behavior of control groups (CTRL) without female mice in chambers demonstrates the effectiveness of the adapted method. (**c**) represents the average heatmap visualization of the nose-point track. Control mice mostly demonstrate locomotor and exploratory activity over the whole cage compared to other groups. Full graphics are presented in Supplementary Materials. Data are mean ± SEM.

Figure 2a,b demonstrate the most critical parameters of the SIMT test. The control group (CTRL) without female mice in chambers shows that in the absence of sexual incentive, male mice have no place preference within the experimental area (Figure 2c). There was a statistically significant effect of the presence of females on the number of visits to the female zone (Figure 2c) and the percent of the time in the nose zone (Figure 2b) of WT mice (F (1, 107) = 20.93, *p* < 0.0001 and F (2, 167) = 26.81, *p* < 0.0001, respectively).

**Figure 3:**
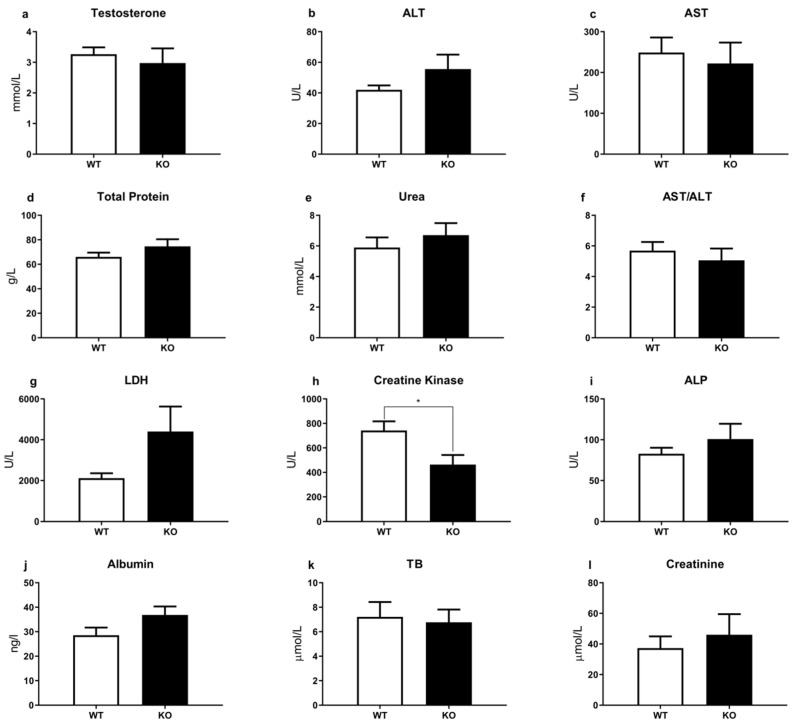
Comparative analysis of basic biochemical and hormonal parameters in the blood of TAAR1-KO and WT mice. (**a**) Testosterone, (**b**) alanine aminotransferase (ALT), (**c**) aspartate aminotransferase (AST), (**d**) total protein, (**e**) urea, (**f**) de Ritis ratio (AST/ALT), (**g**) lactate dehydrogenase (LDH), (**h**) creatine kinase, (**i**) alkaline phosphatase (ALP), (**j**) albumin, (**k**) total bilirubin (TB), (**l**) creatinine. The biochemical screening did not reveal significant differences in any of the demonstrated parameters. Only the creatine kinase comparative analysis shows minimal alterations (WT = 741.7 ± 75.02; KO = 463.5 ± 78.47). Data are mean ± SEM. * *p* < 0.05.

In the presence of sexual incentive, there are no significant differences in the number of visits to the female zone (Figure 2a) and the percent of the time in the nose zone (Figure 2b), between WT and TAAR1-KO mice (F (1, 115) = 1.246, *p* = 0.2666 and F (1, 115) = 1.254, *p* = 0.2651, respectively). All other sexual motivation behavioral parameters also revealed minimal alterations. The complete results of the SIMT tests are presented in the Supplementary Materials (Figure S1). In addition, the blood testosterone analysis (Figure 3a) did not reveal significant differences between WT and TAAR1-KO mice.

### 3.2. TAAR1 Gene Knockout Does Not Significantly Affect Biochemical Parameters

**Figure 4:**
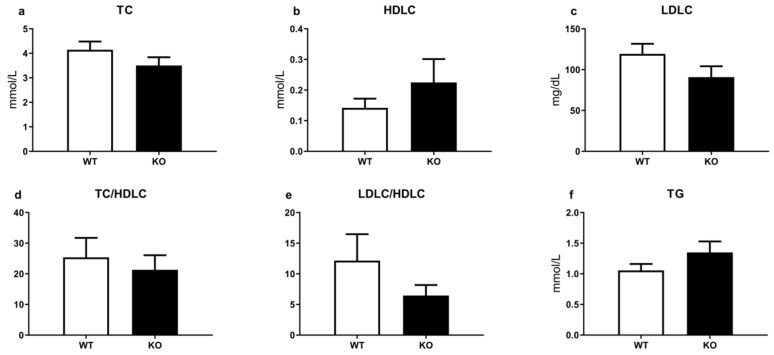
Comparative analysis of main lipid exchange biochemical parameters and ratios in the blood of TAAR1-KO and WT mice. (**a**) Total cholesterol (TC), (**b**) high-density lipoprotein cholesterol (HDLC), (**c**) low-density lipoprotein cholesterol (LDLC), (**d**) total cholesterol/high-density lipoprotein ratio (TC/HDLC), (**e**) ratio of low-density lipoprotein cholesterol and high-density lipoprotein cholesterol (LDLC/HDLC), (**f**) triglycerides (TG). There are only minimal alterations in all presented parameters. Data are mean ± SEM.

The comparative analysis of TAAR1 and WT did not reveal significant differences in major biochemical parameters, such as alanine aminotransferase (ALT), aspartate aminotransferase (AST), total protein, urea, triglycerides (TG), lactate dehydrogenase (LDH), alkaline phosphatase (ALP), total cholesterol (TC), low-density lipoprotein cholesterol (LDLC), high-density lipoprotein cholesterol (HDLC), albumin, total bilirubin (TB), creatinine (Figure 3b and Figure 4). Only creatinine kinase levels were significantly decreased in mutant mice (Figure 3h).

## 4. Discussion

In the present study, we adapted a SIMT behavioral test for mice and evaluated the effect of TAAR1 gene deletion on sexual motivation and testosterone levels. Furthermore, we assessed hematological parameters with a routine biochemistry screening panel. TAAR1-based therapies have a strong potential in the treatment of several human disorders such as schizophrenia, addiction, depression, diabetes, and obesity [4,5]. TAAR1 agonists have already entered phase III clinical trials to treat schizophrenia [7]. Therefore, it is essential to consider the potential side effects of TAAR1-based therapies and preliminarily evaluate the safety profile in the periphery.

There are no commonly accepted protocols concerning non-contact sexual motivation and social recognition that have been reliably established for mice. Therefore, we had to integrate several features from the existing rat test paradigm [45]. Classic tests often use additional males as social recognition validation objects. However, the social hierarchies of rodents are regulated by odor and sniffing interactions [46]. Furthermore, rodents are sensitive to other male odor stimuli, and the existence of alpha males in the experimental arena may affect sexual motivation and lead to additional social stress [47]. Thus, only male and female mice socially interacted through the cage in our experiment. It should be noted, however, that the lack of male conspecifics does not exclude the possibility that the female was approached not only due to sexual motivation, but also due to social interaction or a combination of these. Moreover, the arena parameters were changed to accommodate the smaller size of mice. Finally, we performed a fast screening neurobiological test, which allows one to evaluate sexual motivation in mice quickly. The adapted SIMT method can be used in future pharmacological experiments and as a preliminary step in copulatory sexual tests.

While TAAR1-KO mice demonstrated minimal alterations in sexual motivation, further studies of this kind in the TAAR field are warranted. Recent studies revealed TAAR5 localization in the glomerular and deeper olfactory bulb layers projecting to the limbic brain olfactory circuitry [21]. It is well established that sexual behavior is regulated in mice via the olfactory system [48], and the TAAR5 agonist trimethylamine is considered a pheromone in mice [49]. Moreover, TAAR5 knockout mice show increased adult neurogenesis and a higher number of dopamine neurons [22]. It is likely that other “olfactory” TAARs (TAAR2-TAAR9) are also similarly involved in the transmission of innate odors into limbic brain areas regulating emotions and adult neurogenesis [23]. Thus, the evaluation of the role of TAARs in sexual behaviors may become a prospective direction for future studies.

The analysis of testosterone and other routine blood biochemical parameters in TAAR1-KO and WT mice also demonstrated minimal alterations. Only creatine kinase showed a significant decrease in mutant mice. Such changes may be related to an increased locomotor activity in TAAR1-KO mice [50]. These observations indicate that despite the known expression of TAAR1 in pancreatic β-cells, the stomach, the intestines, the thyroid gland, and leucocytes, the lack of TAAR1 minimally affects lipid and metabolic processes in normal conditions. Potentially, TAAR1-mediated non-canonical mechanisms in the periphery could be revealed under pharmacological or specialized diet challenge conditions.

In conclusion, even such a drastic manipulation as the elimination of TAAR1 did not cause significant alterations in sexual or social motivation, testosterone levels, and blood biochemical parameters. These observations suggest that future TAAR1-based therapies will likely have a good safety profile.

## Data Availability

Data will be available upon request from the corresponding author.

## Supplementary Materials

The following supporting information can be downloaded at: https://www.mdpi.com/article/10.3390/brainsci12050614/s1, Figure S1: Comparative SIMT analysis of TAAR1-KO and WT mice; Figure S2: Additional parameters of SIMT test; Table S1: Comparative biochemical and hormonal analysis of TAAR1-KO and WT mice.
