# Supplementary material for: Evaluation of Approach to a Conspecific and Blood Biochemical Parameters in TAAR1 Knockout Mice
Source: Brain Sci. 2022 May 8;12(5):614. doi: 10.3390/brainsci12050614 (PMC9139149; doi:10.3390/brainsci12050614)
Supplement: Supplementary file 1 [file brainsci-12-00614-s001.zip › brainsci-1657998-supplementary.pdf]

Supplementary Materials

A.

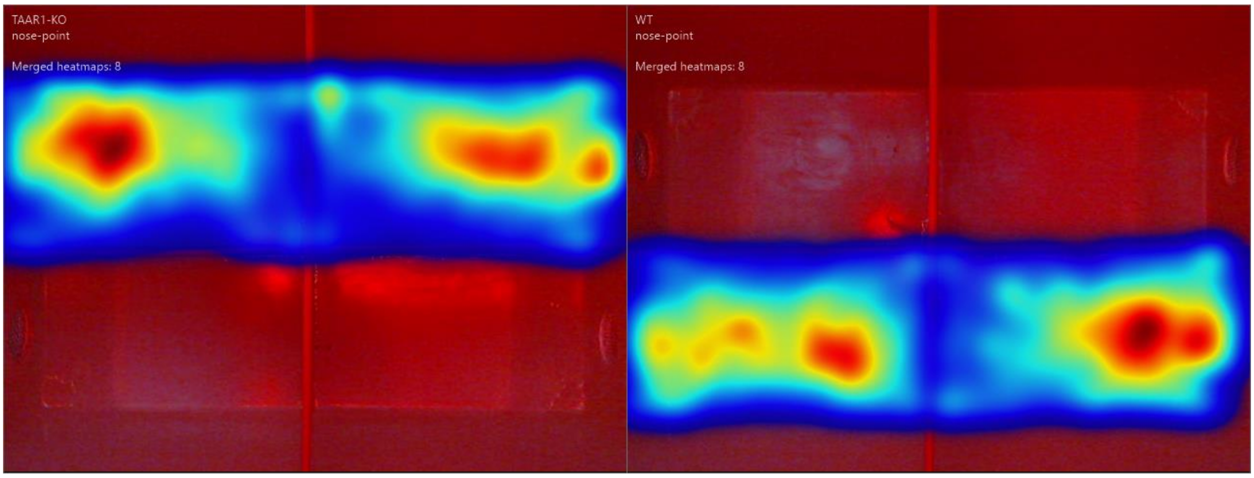

B.

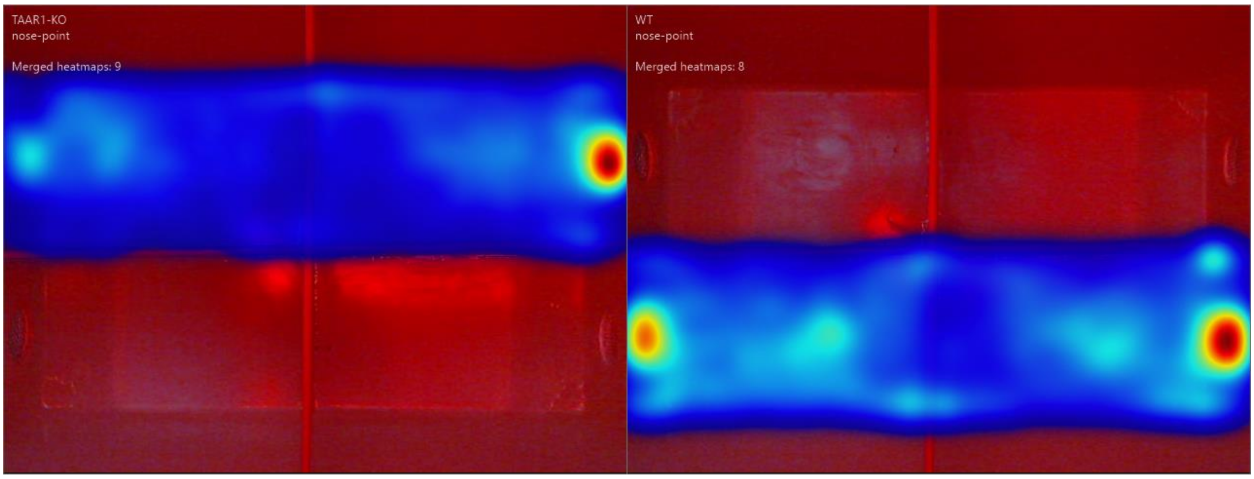

Figure S1. Heatmap visualization of comparative SIMT analysis of TAAR1-KO and WT mice. (Heatmap visualization of movement for TAAR1 and WT in SIMT experiment.)

There are significant differences in nose-point detection trajectories (n=15). In Figure S1 A presented experiment without female mice in cages. Thus, we can observe mostly investigational locomotors activity. In Figure S1 B male mice a mostly attracted by female estrus stimulus. Such heatmap visualization additionally supports effectiveness of adapted sexual motivation method for mice. Data are mean ± SEM.

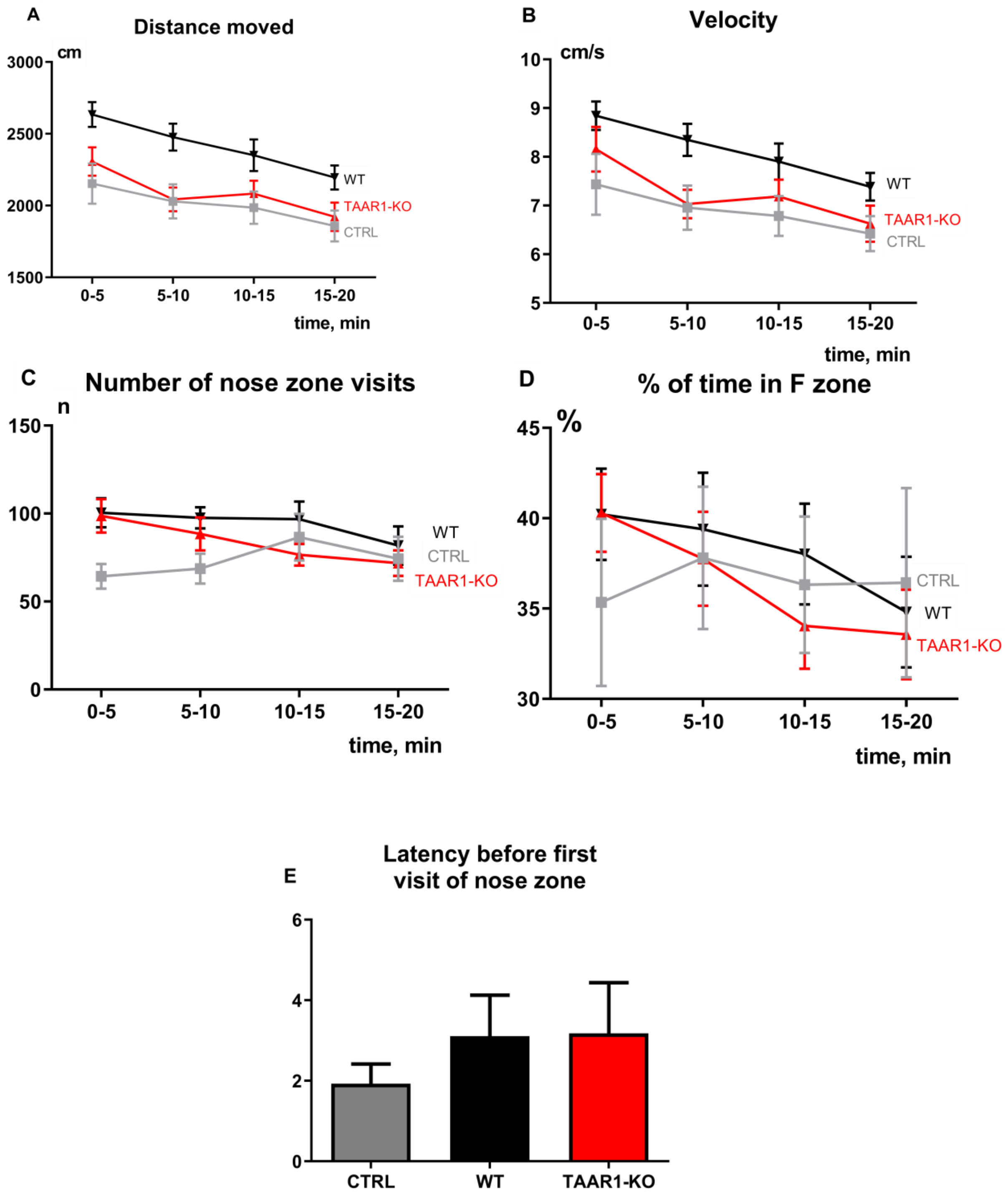

Figure S2. Additional parameters of SIMT test. Control wild type group without female (CTRL, n=15), wild type control group (WT, n=15), TAAR1 homozygous knock-out group, (TAAR1-KO, n=15). Data are Mean ± SEM. There are no significant differences in additional parameters were found (Figure S2 A-E). Data are mean ± SEM.

Table S1. Comparative biochemical and hormonal analysis of TAAR1-KO and WT mice (Data are Mean ± SEM).

Parameters (units)	WT	TAAR1-KO	Number of samples	Dilution factor
Testosterone (mmol/L)	3.26 ± 0.23	2.98 ± 0.48	WT = 8; KO = 7	3
ALT (U/L)	42 ± 2.87	55.57 ± 9.45	WT = 9; KO = 7	5
AST (U/L)	248.8 ± 37.11	222 ± 51.28	WT = 9; KO = 7	5
TP (fL)	66 ± 3.57	74.63 ± 5.86	WT = 14; KO = 13	10
Urea (mmol/L)	5.9 ± 0.66	6.7 ± 0.79	WT = 8; KO = 7	5
TG (g/dL)	1.06 ± 0.105	1.35 ± 0.18	WT = 9; KO = 8	5
LDH (U/L)	221.8 ± 23.8	440.1 ± 122.8	WT = 8; KO = 7	5
Creatine Kinase (U/L)	741.7 ± 75.02	463.5 ± 78.47	WT = 9; KO = 7	10
ALP (U/L)	82.78 ± 7.41	100.8 ± 18.74	WT = 9; KO = 7	5
TC (mmol/L)	4.14 ± 0.34	3.5 ± 0.34	WT = 7; KO = 6	5
LDLC (mg/dL)	119.3 ± 12.46	90.83 ± 13.32	WT = 7; KO = 6	5
HDLC (mmol/L)	0.14 ± 0.03	0.23 ± 0.08	WT = 7; KO = 6	5
Albumin (ng/L)	0.35 ± 0.05	0.22 ± 0.04	WT = 7; KO = 6	5
Total Bilirubin (μmol/L)	7.21 ± 1.21	6.77 ± 0.01	WT = 7; KO = 6	5
Creatinine (μmol/L)	37.29 ± 7.68	46.01 ± 13.47	WT = 7; KO = 6	5
